# Links between oral diseases and metabolic syndrome: a narrative review

**DOI:** 10.1038/s41415-025-8324-0

**Published:** 2025-06-13

**Authors:** Hibah Syed, Simran Sehra, Samira Farzadi, Sadia Niazi

**Affiliations:** 631466143814337910523 King´s College London, London, United Kingdom; 390481805778619922065 Department of Endodontics, Centre of Oral, Clinical and Translational Sciences, Faculty of Dentistry, Oral and Craniofacial Sciences, Guy´s Dental Hospital, King´s College London, London, United Kingdom

## Abstract

**Context** Metabolic syndrome is a group of conditions that involves insulin resistance, hypertension and low high-density lipoproteins; all of which increases a patient's risk of developing cardiovascular diseases. With the prevalence of metabolic syndrome on the rise, researchers have explored the potential for its association with oral diseases, such as periodontitis, apical periodontitis, peri-implantitis, caries and oral potential malignant disorders.

**Objectives** This narrative review explores and collates the current evidence regarding the complex pathophysiology of metabolic syndrome and its associations with oral diseases.

**Current knowledge and gaps in research** It is widely known within the scientific community that oral and systemic diseases can be associated, often with a bidirectional link. Metabolic syndrome and a number of oral diseases are characterised as inflammatory conditions and hence the most well-studied plausible pathway lies within the pro-inflammatory response. Although some oral diseases have an established association, other conditions have not been explored in depth. While the direction of this research seems to be positive, there must be further qualitative longitudinal studies to determine causality. Most importantly, there must be an increased focus on population-wide education to relay the importance of oral health maintenance in association to systemic diseases.

**Conclusion** This narrative review highlights the existing evidence about metabolic syndrome links with oral diseases, while outlining the gaps in the available evidence and direction of future research and population-wide education.

## Key points

- Explores the strength of association between oral health and systemic health.
- Discusses the need for population-wide education to prioritise oral health.
- Investigates the rapidly increasing rate of metabolic syndrome.
- Suggests future research proposals to strengthen existing data.

## Metabolic syndrome

Metabolic syndrome (MetS) refers to a collection of features, including insulin resistance associated with high waist circumference, hypertension and low high-density lipoproteins (HDL), all of which are interconnected and pose as risk factors for type 2 diabetes mellitus and cardiovascular disease. The definition of MetS has been contentious within the clinical and scientific communities but there are four main definitions (Table 1).^1^^,^^2^

**Table 1:** A table showing the various assessment criteria of MetS

	**World Health Organization (1999)3**	**The European Group for Study of Insulin Resistance (1999)4**	**National Cholesterol Education Program Adult Treatment Panel III (2001)5**	**International Diabetes Federation (2005)6**
Absolutely required	Insulin resistance	Hyperinsulinaemia: plasma insulin > 75th percentile	None	Central obesity waist circumference: ≥94 cm M, ≥80 cm F
General criteria	Insulin resistance/T2D and 2/5 criteria below	Hyperinsulinaemia + 2/4 criteria below	3/5 criteria below	Obesity + 2/4 criteria below
Obesity measure	Waist-to-hip ratio: >0.90 (M), >0.85 (F), or BMI >30 kg/m2	Waist circumference: ≥94 cm (M), ≥80 cm (F)	Waist circumference: >101.6 inches (M), >88.9 inches (F)	Obesity is already required
Glucose levels	Insulin resistance is already required	Insulin resistance is already required	Fasting glucose: ≥100 mg/dl	Fasting glucose: ≥100 mg/dl
Cholesterol	TG ≥150 mg/dl or HDL-C	TG ≥177 mg/dl or HDL-C	TG ≥150 mg/dl or Rx	TG ≥150 mg/dl or Rx
Hypertension (high blood pressure)	≥140/90 mmHg	≥140/90 mmHg	>130 mmHg systolic, >85 mmHg diastolic	>130 mmHg systolic, >85 mmHg diastolic

The first of these was established by World Health Organization (WHO) in 1999. It highlighted insulin resistance as the primary pathophysiology of MetS and is an essential requirement for its diagnosis. According to this, the insulin resistance was measured in three main ways: impaired fasting glucose; impaired glucose tolerance; or via an elevated homeostatic model assessment of insulin resistance, which takes into account the proportionality between fasting insulin and fasting glucose. According to this definition, two additional criteria from hypertension, dyslipidaemia, microalbuminuria and obesity must be met in order to be diagnosed as MetS. The measurements for obesity were using a waist-to-hip ratio and through body mass index (BMI).^3^

Similarly, the European Group for the Study of Insulin Resistance (EGIR) centres insulin resistance in MetS pathophysiology but uses an alternative definition.^4^ EGIR characterises insulin resistance as ‘a fasting plasma insulin value that is greater than the 75th percentile' (1999).^4^ This acts as an easier diagnostic tool; however, it fails to consider type 2 diabetics (as fasting insulin is not an appropriate measurement), thus making this definition somewhat over-simplistic. Furthermore, much like the WHO, EGIR requires the patient to have hypertension, dyslipidaemia or obesity (measured by waist circumference) in order to meet the criteria for MetS.^4^

One of the most commonly used definitions are by the National Cholesterol Education Program (NCEP-ATP [Adult Treatment Panel] III) (2001).^5^ Unlike the previous definitions by WHO and EGIR, patients with MetS are not required to have insulin resistance. Rather, three or more of five criteria are required to be diagnosed with MetS. The five criteria include: ‘waist circumference over 40 inches for men and 35 inches for women, blood pressure over 130/85 mmHg, fasting triglyceride (TG) level over 150 mg/dl, fasting HDL cholesterol (HDL-C) level less than 40 mg/dl for men or 50 mg/dl for women, and fasting blood sugar over 100 mg/dl'.^5^ This definition has categories that can be easily measured and assessed by clinicians and interlinks the categories required for metabolic syndrome (insulin resistance, obesity, low levels of HDL and elevated levels of low-density lipoproteins, and high blood pressure) without assuming that the main cause of metabolic syndrome is insulin resistance, as done by the WHO and EGIR definition.^5^

An updated definition of MetS was coined by the International Diabetes Foundation in 2005.^6^ This also considered how a patient's ethnicity and nationality have an effect on their risk of developing metabolic disease. For example, the South Asian population have a higher risk of developing cardiovascular disease and type 2 diabetes in comparison the Western population. Therefore, a lower hip measurement is required to classify a South Asian patient with metabolic syndrome than for a patient from a Western population. In this definition, obesity is required for a person to have metabolic syndrome, measured by waist circumference – more than or 94 cm for men and more than or 80 cm for women. Two of the four additional criteria must also be met from the high fasting glucose level, high lipid level, low HDL level and hypertension.^6^

## Link between metabolic syndrome and periodontitis

Periodontitis has an established link to several systemic diseases. Emerging research showed that patients with MetS were twice more likely to suffer from periodontitis.^7^ This suggests that there is a significant epidemiological association between the two conditions.^7^^,^^8^ Periodontitis is a chronic inflammatory condition triggered by the host-microbiome interactions between the dysbiotic subgingival microbial biofilm and impaired host immune response.^9^^,^^10^^,^^11^^,^^12^^,^^13^ This chronic inflammation causes extensive damage to the surrounding tissues of the tooth, leading to tooth attachment loss. The evidence suggests that along with the dysbiotic subgingival microbial biofilm, a patient's genetic predisposition can also lead to higher risk of development of severe periodontitis. Furthermore, there is a depth of evidence to suggest that the pathology of periodontitis is initiated by surrounding environmental factors, such as increased oxidative stress and systemic inflammation creating a dysbiosis in the oral environment. This is seen most markedly with type 2 diabetes due to high blood glucose causing the release of advanced glycation end products, which interact with receptors to trigger an immune response, resulting in gingival inflammation, clinical attachment and bone loss.^10^

Dysbiosis and a dysfunctional host response can also be caused by other factors of MetS, for example, studies have shown that the relation between periodontitis and MetS can be due to their common risk factors. The primary aetiology of MetS is thought to be via the pro-inflammatory response, which can occur as a result of insulin resistance: a condition characterised by inefficient binding of insulin to fat, muscle and liver cells, resulting in poor glucose uptake.^14^ Specifically, increased presence of inflammatory cytokines, such as C-reactive protein and interleukin-6, significantly increase insulin resistance. Both conditions exhibit inflammation as a common pathway, whereby the disease severity is positively correlated with the extent of inflammation.^7^ This theory has been substantiated by another study which showed a positive association between periodontitis and MetS, with a 95% confidence level.^15^ The cross-sectional study showed that the prevalence of MetS and periodontitis was 67.06% and 55.37%, respectively.^15^ The mechanism of inflammation has also been explored here, suggesting that the dissemination of bacteria occurs as a result of periodontitis, leading to chronic systemic inflammation which increases the risk of MetS. However, much like other studies, limitations include small sample size and observational data, which hinder the ability to determine causation.^15^

There is also evidence to suggest that the association between periodontitis and MetS may be linked to oxidative stress as a result of the pro-inflammatory response. There is a biologically plausible pathway that shows an increased serum level of products related to oxidative stress in impacted periodontal tissues, causing an imbalance of reactive oxygen species, which also increases insulin resistance.^14^^,^^16^

The bi-directional effect of the two conditions has been explored in cross-sectional studies and one of which suggests that the presence of MetS acts as a predisposing factor for periodontitis. For example, patients with MetS often experience central obesity and therefore have increased levels of adipose tissue. Adipose tissues contribute to the production of prostaglandins and cytokines which directly contribute towards the progression of periodontitis.^17^

While there is still contention surrounding the exact pathway and relationship between MetS and periodontitis, highlighting the need for more longitudinal studies, existing evidence suggests the association should not be ignored.^7^ The prevalence of periodontitis being 37% in the United Kingdom (UK), coupled with the rising diagnosis rate of MetS, suggests that future research may be potent for clinical practice.^18^

## MetS and apical periodontitis

There have been several studies which display an association between periodontitis and patients with MetS, with the severity and prevalence of periodontitis being greater in patients with MetS than without.^19^ However, there is yet to be more research conducted to confirm a relationship between the prevalence of MetS and apical periodontitis.

Apical periodontitis is the body's host response to an infected pulp, resulting in localised inflammation in the periapical area of the tooth. The presence of pathogenic microorganisms in the root canal can trigger this immune response. Apical periodontitis may be symptomatic or asymptomatic but its early diagnosis is important to prevent progression of the disease. Treatment is generally root canal treatment, root canal re-treatment, apical surgery, or extraction.^20^

A recent cross-sectional study aimed to determine the association between patients with apical periodontitis and cardiovascular disease, as well as the prevalence of patients with apical periodontitis and metabolic syndrome.^21^ The study had two groups of participants, one with cardiovascular events and the other with healthy subjects. The total dental index and periapical index was used to determine the level of oral inflammation and the presence of apical periodontitis, respectively. It was found that the prevalence of apical periodontitis is much higher in patients with cardiovascular events and the oral health of these patients are poorer. Apical periodontitis and oral inflammation were both associated with cardiovascular events. In regard to the relationship between apical periodontitis and MetS, patients with MetS had poorer oral health diagnosed by the modified total dental index score but there was not a significant effect on periapical health of teeth or the number of root-filled teeth. Patients with MetS had more oral inflammation but not apical periodontitis specifically. It has been suggested that the link between oral inflammatory conditions and MetS is through the modulation of leptin levels.^21^

A study done among middle-aged, Chinese participants showed an association between MetS and caries, with MetS patients having a higher prevalence of caries.^22^ Caries is one of the possible aetiological factors for apical periodontitis, so this link can be explored further in the future.^23^ Links have also been made between apical periodontitis and components of MetS; a study on rats showed that lipid levels were affected by the induction of apical periodontitis.^24^

In a 2022 study, which determined the impact of apical periodontitis on the presence of cardiovascular disease risk biomarkers, it was found that patients with apical periodontitis had significantly higher serum levels of high-sensitivity C-reactive protein, asymmetric dimethylarginine and matrix metalloproteinase-2, along with higher MetS factors compared to the healthy controls.^25^ When reviewed at one year and two years after non-surgical root canal re-treatment and apical surgery, not only was good apical healing achieved, but these inflammatory biomarker levels and MetS factors also significantly reduced compared to the baseline values.^26^ This recent study outlined the systemic inflammatory burden that occurs as a result of un-treated apical periodontitis and also raises the MetS indicators, whereas the successful endodontic treatment of chronic apical periodontitis results in improvements of these metabolic syndrome indicators, including better glycaemic control.^25^^,^^26^ This study acts a significant cornerstone in research as it is the first, largest, longitudinal study with a two-year recall, which enables researchers to assess the impact of endodontic treatment on MetS. This, in turn, elucidates the strength of association between apical periodontitis and metabolic disease.

Despite promising research, further longitudinal studies must be conducted to draw a firm conclusion and a possible bidirectional link between MetS and apical periodontitis.

## Link between MetS and oral potential malignant disorders

The association between MetS and oral health manifestations extends to oral potential malignant disorders (OPMD), which has been shown through various cross-sectional studies.^27^^,^^28^ There are varying types of OPMD that may occur, for example, leukoplakia, proliferative verrucous leukoplakia, erythroplakia, oral submucous fibrosis, oral lichen planus, actinic keratosis (actinic chellitis), palatal lesions in reverse smokers, oral lupus erythematosus, dyskeratosis congenita, oral lichenoid lesion, and oral graft versus host disease. The presence of OPMD is associated with a statistically increased risk of developing oral cancers throughout the patient's lifetime; however, only a minority progress to cancer. Clinically, they present with varying features topographically, as well as in colour and size, with some lesions also presenting with ulcerations and erosive lesions.^27^^,^^28^^,^^29^

A study in Taiwan found that oral cancer had the fourth-highest mortality rate of cancer deaths of men aged 25–44.^30^ One of the lifestyle factors that increases the likelihood of the development of these diseases is betel nut chewing, which is significant in South Asia. The study found that MetS in betel nut chewers was significantly associated with oral pre-malignancy after adjusting for the confounding factors of age, sex, betel nut chewing, smoking and alcohol drinking.^30^ These findings were in alignment with other research which suggest that OPMD had a significant association to the manifestations of metabolic disease, which consist of high blood pressure, central obesity and high blood glucose.^31^^,^^32^

It has been theorised that the biological mechanism occurring is through the effects of insulin resistance which cause a dysregulation in lipid signalling, thus altering the levels of inflammatory markers and cytokines. In turn, the activation of these deregulatory pathways causes a pre-disposition to ‘haematologic malignancies' resulting in chemo-resistance.^33^

There should be a more concerted effort to create health programmes that view oral health through the lens of metabolic syndrome as it is a collection of symptoms that are relatively common among the UK population. Data have shown 5% of adolescents, globally, suffer from MetS, a figure likely to increase unless early prevention is established.^34^

## Metabolic syndrome and caries

Dental caries is ‘a biofilm-mediated, sugar-driven, multifactorial, dynamic disease that results in the phasic demineralisation and remineralisation of dental hard tissues'.^35^ A lesion occurs when there is more demineralisation than remineralisation.^36^

Over the last few decades, there has been a shift towards a Westernised diet, which includes an increase in the consumption of carbohydrates, which encourage pathological changes in the oral microbiota, including increased representation of acid-producing and acid-tolerant organisms.^37^ This has shifted the oral microbiome and oral heath haemostasis and resulted in increased levels of dental caries among the population.^38^ This shift has not only given rise to oral diseases but also systemic diseases, such as diabetes and MetS.

There have been a few studies that have analysed the association, if any, between MetS and caries and there have been varying outcomes. A recent systemic review and meta-analysis narrowed down research to 13 studies which met their criteria and assessed the link between oral hygiene and MetS. They found that interdental brushing and toothbrushing corresponded with a lower risk of MetS but more research needs to be conducted to establish a significant association between the number of visits to the dentist and MetS.^39^

Another study was conducted which explored the relationship between MetS and caries and it included 13,998 middle-aged, Chinese participants. Each patient was screened for MetS and the number of decayed, missing and filled teeth were calculated. It was found that the patients who had caries were more likely to have MetS but after adjustment of results and stratification, they discovered that different components of MetS held varying significance in relation to caries. There was no significant relation between elevated lipid levels, obesity, low HDL, hypertension and caries. There was, however, a relationship between caries, MetS and hyperglycaemia.^40^ This study was independent to tooth loss, periodontal health and health behaviours.

A study evaluating the relationship between diabetes and caries which compared the caries index in two groups of participants – one group with participants who have diabetes and caries and the other group who have no systemic disease but have caries – found that there was a higher level of *Streptococcus mutans* in patients with diabetes. This was suggested to be due to the reduced salivary flow in patients with diabetes. Saliva acts as a buffer and protects teeth.^41^ There still, however, needs to be larger and more comprehensive studies conducted on MetS and the prevalence of caries.

## Metabolic syndrome and peri-implantitis

Peri-implantitis is a disease affecting dental implants in which bone resorption around the implant takes place due to inflammatory processes and osteoclasts. It is a leading cause of failure for implants. Inflammatory mediators and markers that have been found present in cases of peri-implantitis are also associated with components of MetS, such as diabetes mellitus. Therefore, a link could be established between the two conditions and if they are exacerbated by each other.^42^

From a literature review which targeted papers about MetS and peri-implant disease, the results were narrowed down to six studies. There were no studies directly linking the two and any associations made to cardiovascular disease were inconsistent. People with hyperglycaemia were found to be at greater risk of peri-implantitis.^43^

In a cross-sectional study, the metabolic status was evaluated for 183 participants who had at least one dental implant. MetS was diagnosed using the NCEP-ATP III criteria.^5^ Out of the patients who had MetS, peri-implantitis was diagnosed in 36.9%, compared to 26.3% of patients without MetS who had peri-implantitis. However, further studies and research is required to establish a relationship between the two.^44^

In 2023, a systematic review was conducted in order to address the question: ‘what is the effect of peri-implant diseases on metabolic syndrome factors?'.^45^ After defining the criteria, searching for studies and the bias assessment (using the Newcastle-Ottowa scale), five studies were included in the review, all of which were cross-sectional. The review suggested that although there are some links between components of MetS and peri-implantitis, no direct association can be made at this stage without stronger evidence. Out of the five cross-sectional studies, one concluded that peri-implantitis resulted in an increased level of serum lipids, but another found no significant change in TG levels in patients with peri-implant mucositis compared to participants with implant health or peri-implantitis. The review acknowledges evidence that is suggestive of subjects with hyperglycaemia having an increased chance of developing peri-implantitis than those without. The suggested mechanism is the reduced immune response and decreased blood supply in hyperglycaemic patients. This is suggestive of a bi-directional association between MetS and peri-implantitis. Some studies did not show a significant association between blood sugar levels and peri-implantitis but study size was small and factors such as smoking were not controlled. Overall, although there is increasing studies being undertaken to determine a direct link between MetS components and peri-implantitis, we would benefit from longitudinal studies with large sample sizes in order to reaffirm a causative relationship between the two.^45^

## Conclusion

Despite emerging evidence highlighting a strong association between MetS and oral health, there is still a depth of research that is required in order to find causative links between MetS and oral health. While cross-sectional studies have innate scientific value, there should be a greater focus on longitudinal studies which follow a set of patients for an extended period of time. This will act as a greater service for researchers and clinicians to understand how MetS is likely to impact oral health throughout a patient's lifetime. This, in turn, enables an adaptation of care for MetS patients, supporting a more positive oral health outcome. This research, if improved, will have a population-wide impact, as it highlights the bidirectional relationship between systemic and oral health, which is often underlooked.
